# Significance of pyroptosis-related gene in the diagnosis and classification of rheumatoid arthritis

**DOI:** 10.3389/fendo.2023.1144250

**Published:** 2023-03-15

**Authors:** Jian Li, Yongfeng Cui, Xin Jin, Hongfeng Ruan, Dongan He, Xiaoqian Che, Jiawei Gao, Haiming Zhang, Jiandong Guo, Jinxi Zhang

**Affiliations:** ^1^ Department of Orthopaedics, Hangzhou Ninth People’s Hospital, Hangzhou, Zhejiang, China; ^2^ Department of Orthopaedics, The First Affiliated Hospital of Zhejiang University of Chinese Medicine, Hangzhou, China

**Keywords:** rheumatoid arthritis, pyroptosis, immunity, consensus clustering, bioinformatic analysis

## Abstract

**Background:**

Rheumatoid arthritis (RA), a chronic autoimmune inflammatory disease, is often characterized by persistent morning stiffness, joint pain, and swelling. Early diagnosis and timely treatment of RA can effectively delay the progression of the condition and significantly reduce the incidence of disability. In the study, we explored the function of pyroptosis-related genes (PRGs) in the diagnosis and classification of rheumatoid arthritis based on Gene Expression Omnibus (GEO) datasets.

**Method:**

We downloaded the GSE93272 dataset from the GEO database, which contains 35 healthy controls and 67 RA patients. Firstly, the GSE93272 was normalized by the R software “limma” package. Then, we screened PRGs by SVM-RFE, LASSO, and RF algorithms. To further investigate the prevalence of RA, we established a nomogram model. Besides, we grouped gene expression profiles into two clusters and explored their relationship with infiltrating immune cells. Finally, we analyzed the relationship between the two clusters and the cytokines.

**Result:**

CHMP3, TP53, AIM2, NLRP1, and PLCG1 were identified as PRGs. The nomogram model revealed that decision-making based on established model might be beneficial for RA patients, and the predictive power of the nomogram model was significant. In addition, we identified two different pyroptosis patterns (pyroptosis clusters A and B) based on the 5 PRGs. We found that eosinophil, gamma delta T cell, macrophage, natural killer cell, regulatory T cell, type 17 T helper cell, and type 2 T helper cell were significant high expressed in cluster B. And, we identified gene clusters A and B based on 56 differentially expressed genes (DEGs) between pyroptosis cluster A and B. And we calculated the pyroptosis score for each sample to quantify the different patterns. The patients in pyroptosis cluster B or gene cluster B had higher pyroptosis scores than those in pyroptosis cluster A or gene cluster A.

**Conclusion:**

In summary, PRGs play vital roles in the development and occurrence of RA. Our findings might provide novel views for the immunotherapy strategies with RA.

## 1 Introduction

Rheumatoid arthritis (RA), a chronic autoimmune inflammatory disease, is often characterized by persistent morning stiffness, joint pain and swelling (1). RA affects approximately 1% of the world population and has become one of the most common causes of significant disability (2). Although the pathogenesis and etiology of RA have not been fully known, the interaction of environmental, genetic, and immunological factors has been shown to play an important role in the development of RA (3). Early diagnosis and timely treatment of RA can effectively delay the progression of the condition and significantly reduce the incidence of disability (4). Therefore, screening for diagnostic genes associated with RA, exploring their subtype classification, and elucidating the underlying pathogenesis of RA could be effective in preventing and treating RA, and might provide new approaches for clinical treatment of RA.

Pyroptosis, a novel inflammatory programmed cell death, is mediated by the caspase family and the GSDM protein family (5). Pyroptosis is characterized by cell swelling and cell membrane rupture, and the release of pro-inflammatory cytokines that eventually induce and aggravate the inflammatory response (6). Increasing studies conformed that pyroptosis might play a key role in the development of many immune diseases (7). In the arthritic mouse model, NLRP3^-/-^ or Caspase-1^-/-^ mice could alleviate symptoms of arthritis (8). Gsdme^-/-^ mice have been demonstrated to reduce intestinal inflammation in the inducible colitis model (9). Besides, bronchial epithelial cell pyroptosis promotes airway inflammation in asthmatic mice (10). However, the role of pyroptosis-related genes (PRGs) in RA remains unclear.

In the research, we used bioinformatics methods to investigate the function of PRGs in the diagnosis and classification of rheumatoid arthritis form the Gene Expression Omnibus (GEO) datasets. Firstly, we identified differential expression of PRGs from the GSE93272 dataset. Then, we screened 5 PRGs associated with RA by support vector machine-recursive feature elimination (SVM-RFE), least absolute shrinkage and selection operator (LASSO) logistic regression and random forest (RF) algorithms, and established a nomogram model for predicting the prevalence of RA. In addition, we divided gene expression profiles into two clusters and explored their relationship with infiltrating immune cells. Finally, we further analyze the relationship between two clusters and cytokines. We found that the pyroptosis-related pattern could distinguish RA patients from normal people and provide new directions for the prevention and treatment of RA.

## 2 Materials and methods

### 2.1 Data acquisition and preprocessing

The microarray datasets were downloaded from the GEO database (https://www.ncbi.nlm.nih.gov/geo/) using “rheumatoid arthritis”, “whole blood,” and “Homo sapiens” as keywords. The inclusion criteria were as follows: the whole-genome expression profiling of whole blood of RA patients and healthy control samples was available in the datasets; every dataset contained a sample count of > 20; and all included samples were not treated with drugs. The microarray dataset GSE93272 from the GPL570 platform containing 35 healthy controls and 67 RA patients was downloaded from the GEO database (11).

### 2.2 Identification of differentially expressed PRGs

The GSE93272 cohort was normalized by the “limma” package of R software (12). Based on previous literatures (13–15), we acquired 52 PRGs. However, we did not find the expression data of GSDMA in GSE93272. Therefore, 51 PRGs were used for the following analysis. Then, we identified differentially expressed PRGs in RA and normal samples using the “limma” package. The p-value < 0.05 was considered a significant difference. Heatmap and boxplot were performed using the R packages “pheatmap” and “ggpubr” to visualize the differentially expressed PRGs.

### 2.3 Screening of PRGs for RA

Based on the differentially expressed PRGs, three feature selection algorithms, including SVM-RFE (16), LASSO logistic regression (17) and RF algorithm (18) were adapted to screen RA-related biomarkers, respectively. The SVM-RFE algorithm was performed by the R packages “e1071” and “caret” with five-fold cross-validation (19). The LASSO logistic regression was employed with the R package “glmnet” (20). The RF algorithm was analyzed by the R package “randomForest” (21). Then, the “venn” R package was used to select overlapping genes from the three algorithms as signature genes for further analysis.

### 2.4 Construction of a nomogram model

We constructed a nomogram model based on PRGs (CHMP3, TP53, AIM2, NLRP1, and PLCG1) to predict the occurrence of RA patients with the “rms” package in R (22). The calibration curve was used to assess the predictive performance of the nomogram model. Then, we further performed decision curve analysis (DCA) and clinical impact curve analysis (CICA) to estimate the clinical utility of the nomogram model (23).

### 2.5 Consensus clustering

Consensus clustering is an algorithm for identifying cluster each member and their number in datasets (24). We utilized the consensus clustering method to distinguish distinct pyroptosis-related clinical subtypes of RA and identify different PRGs patterns based on the significant differentially expressed PRGs with the package “ConsensusClusterPlus” in R (25). “Points” represents the score of the corresponding factor below and “Total Points” indicates the summation of all the scores of factors above.

### 2.6 Estimation of the pyroptosis gene signature

To quantify the pyroptosis patterns, we used principal component analysis (PCA) algorithms to calculate the pyroptosis score for each RA sample. The Principal Component 1 (PC1) and Principal Component 2 (PC2) were chosen as the signature scores. And pyroptosis scores for each RA patient were calculated using the following formula (26, 27): Pyroptosis Score = Σ(PC1_i_ + PC2_i_), where i is the expression of PRGs.

### 2.7 Estimation of immune cell infiltration for RA

The single-sample gene-set enrichment analysis (ssGSEA) was employed to measure the relative abundance of immune cells in RA samples *via* the R packages “limma”, “GSVA”, and “GSEABase” (28). And the gene set for marking each immune cell type was obtained from the study of Charoentong (29).

### 2.8 Functional and pathway enrichment analysis

To investigate the functional and molecular pathways of differentially expressed genes between pyroptosis gene clusters A and B, we performed GO, KEGG enrichment analyses by the “colorspace”, “stringi” and “ggplot2” packages in R (30, 31). P < 0.05 was considered statistically significant.

### 2.9 Statistical analysis

The Kruskal-Wallis test was adopted to compare differences between normal samples and RA samples. The significant differences were identified with the p-value < 0.05. All statistical analysis were performed using the R version 4.0.3.

## 3 Results

### 3.1 The landscape of the differentially expressed PRGs

**Figure 1:**
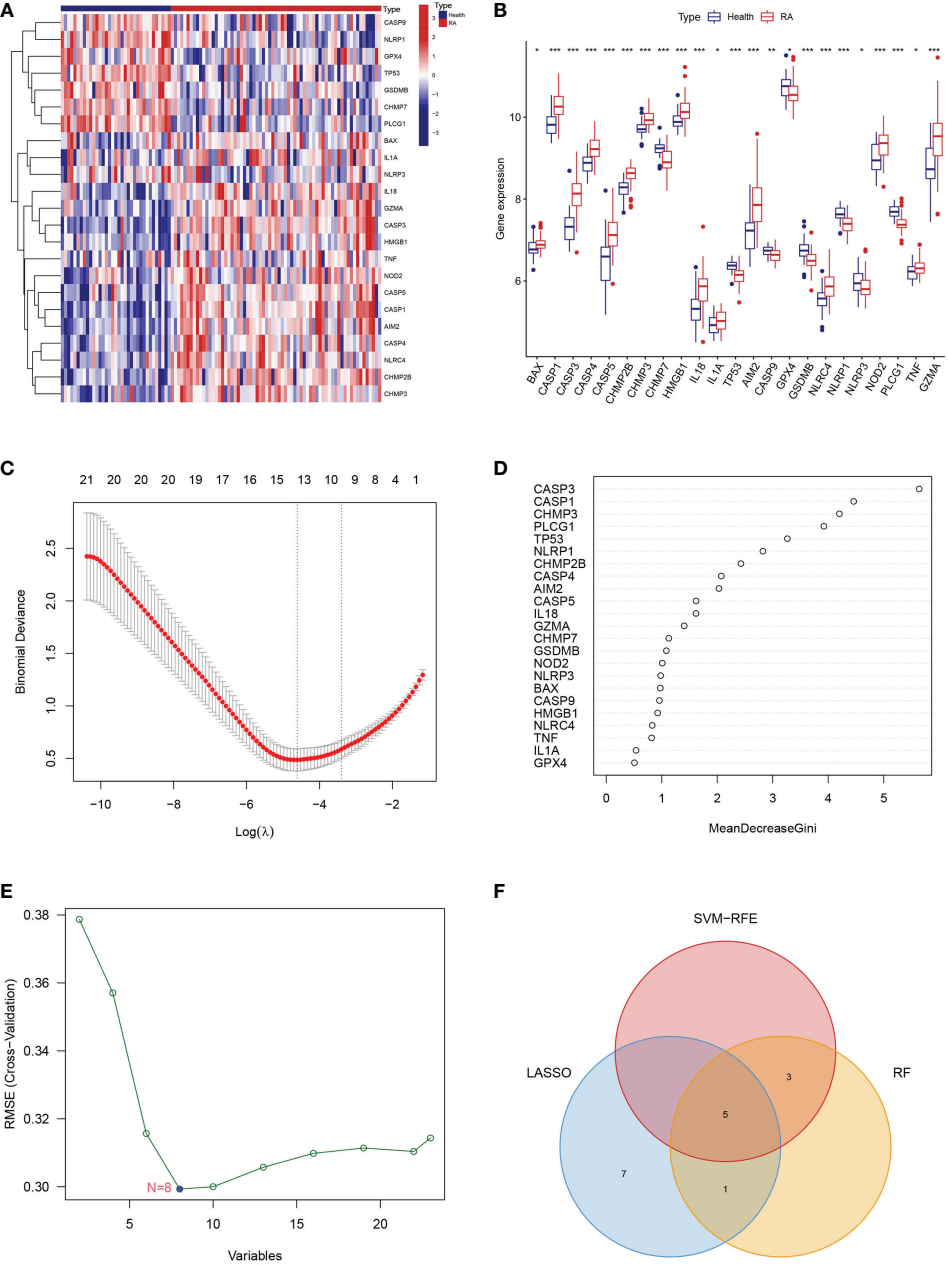
Landscape of the 23 PRGs. **(A)** Expression heatmap of the 23 PRGs in healthy control and RA patients. **(B)** Expression histogram of the 23 PRGs in healthy control and RA patients. **(C)** The PRGs screened using the LASSO logistic regression algorithm. **(D)** The hub genes identified *via* the RF algorithm. **(E)** The PRGs recognized using SVM-RFE algorithm. **(F)** Venn diagram showing the intersection among PRGs genes between the three algorithms. * means P < 0.05, ** means P < 0.01, *** means P < 0.001.

We analyzed the differential expression levels of 51 PRGs between RA patients and healthy controls using the “limma” R package (**Supplementary Table 1**). A heatmap and histogram were used to visualize the 23 differentially expressed PRGs. We found that BAX, CASP1, CASP3, CASP4, CASP5, CHMP2B, CHMP3, HMGB1, IL18, IL1A, AIM2, NLRC4, NOD2, TNF, and GZMA were overexpressed in RA patients compared to healthy controls (**Figures 1A, B**).

### 3.2 Identification of characteristic genes

To further screen the characteristic genes related to PRGs for RA, we utilized the LASSO logistic regression algorithm, the RF algorithm, and the SVM-RFE analysis for feature identification (**Supplementary Table 2**). Thirteen genes from differentially expressed PRGs were identified as biomarkers for RA using the LASSO logistic regression algorithm (**Figure 1C**). We used RF algorithm to detect nine key genes from differentially expressed PRGs as vital biomarkers (**Figure 1D**). Eight signature genes were identified from differentially expressed PRGs by the SVM-RFE analysis (**Figure 1E**). Finally, we overlapped three different algorithms analysis results and obtained 5 genes (CHMP3, TP53, AIM2, NLRP1, and PLCG1) that were significantly related to RA (**Figure 1F**).

### 3.3 Construction of the nomogram

**Figure 2:**
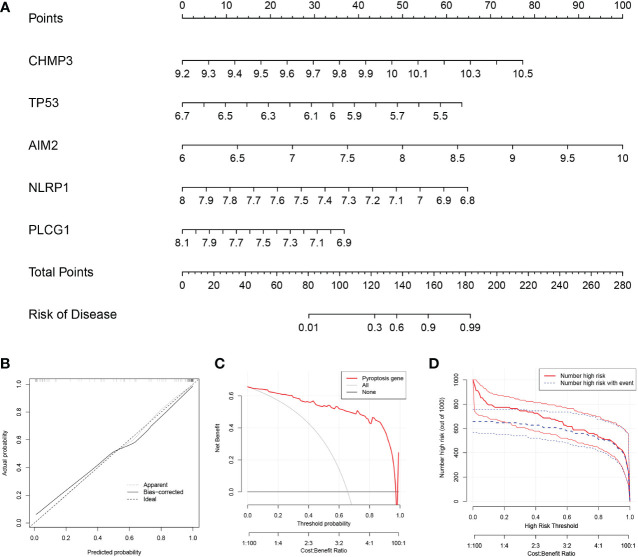
Construction of the nomogram model. **(A)** Construction of the nomogram model based on the 5 PRGs. **(B)** Predictive robustness of the nomogram model as revealed by the calibration curve. **(C)** Decisions based on the nomogram model may benefit RA patients. **(D)** Clinical impact of the nomogram model as assessed by the clinical impact curve.

To predict the prevalence of RA patients, we constructed a nomogram model based on the 5 PRGs (**Figure 2A**). As shown in **Figure 2B**, the calibration curve of the nomogram revealed accurate predictive ability. The DCA result revealed that decision-making based on established models may be beneficial for RA patients (**Figure 2C**). And the CICA result (**Figure 2D**) found that the predictive power of the nomogram model was significant.

### 3.4 Two distinct pyroptosis patterns

**Figure 3:**
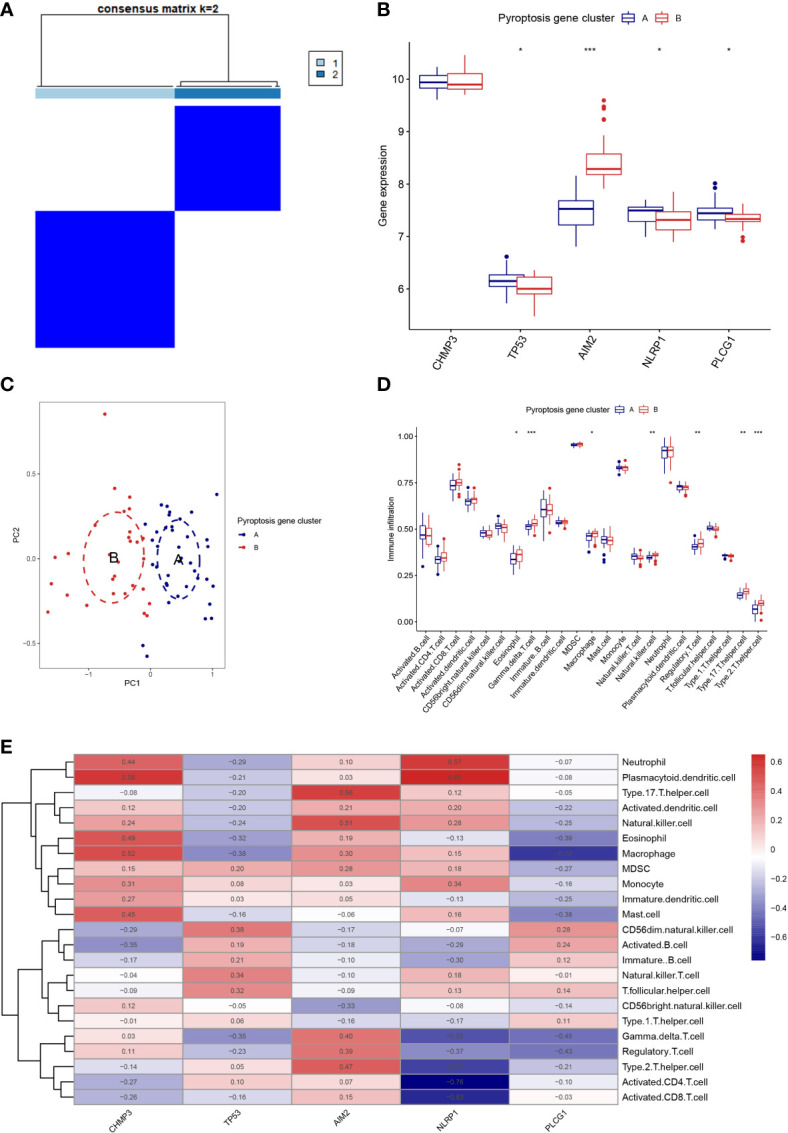
Consensus clustering of the 5 PRGs. **(A)** Consensus matrices of the 5 PRGs for k = 2. **(B)** Differential expression histogram of the 5 PRGs in gene cluster A and B. **(C)** PCA for the expression profiles of the 5 PRGs. **(D)** Differential immune cell infiltration between gene cluster A and B. **(E)** Correlation between infiltrating immune cells and the 5 PRGs. * means P < 0.05, ** means P < 0.01, *** means P < 0.001.

Based on the 5 PRGs, we identified two different pyroptosis patterns (cluster A and cluster B) using the consensus clustering method (**Figure 3A** and **Supplementary Figure 1**). There were 38 cases in cluster A and 29 cases in cluster B. We plotted the histogram to observe the differential expression levels of the 5 PRGs between the two clusters. TP53, NLRP1, and PLCG1 showed higher expression in pyroptosis gene cluster A than in pyroptosis gene cluster B, while AIM2 revealed the opposite results. And CHMP3 showed no differently expressed between the two patterns (**Figure 3B**). As shown in **Figure 3C**, the two pyroptosis patterns could be distinguished though the 5 significant PRGs with PCA analysis. Then, the differential immune cell infiltration between the two pyroptosis patterns was analyzed (**Figure 3D**). We found that eosinophil, gamma delta T cell, macrophage, natural killer cell, regulatory T cell, type 17 T helper cell, and type 2 T helper cell were significant high expressed in cluster B (p < 0.05). Besides, we calculated the abundance of immune cells in RA patients and evaluated the correlation between the 5 PRGs and immune cells (**Figure 3E**).

### 3.5 Function and pathway enrichment

**Figure 4:**
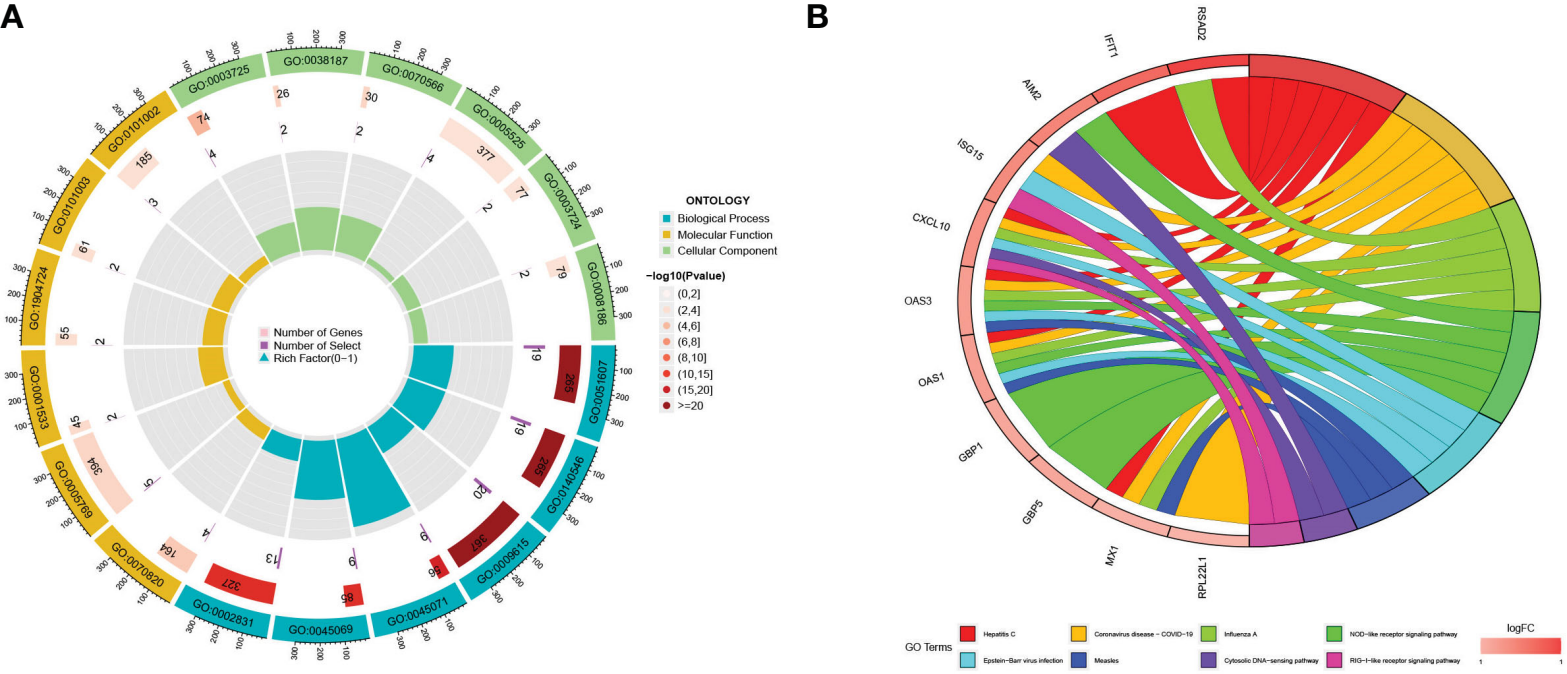
The functional enrichment analyses of DEGs. **(A)** The GO analyses results for DEGs; **(B)** The KEGG analysis results for DEGs.

A total of 56 differentially expressed genes (DEGs) were identified between the two pyroptosis patterns. To further explore the potential functional and molecular pathways of DEGs, we performed GO and KEGG enrichment analyses, and the results were shown through an enrichment circle diagram. In the GO enrichment analysis of differential expression PRGs, biological processes (BP) terms were correlated with defense response to virus (GO:0051607) and defense response to symbiont (GO:0140546); cellular components (CC) terms were related to tertiary granule (GO:0070820) and early endosome (GO:0005769); and molecular functions (MF) terms were associated with double stranded RNA binding (GO:0003725) and pattern recognition receptor activity (GO:0038187) (**Figure 4A**; **Supplementary Table 3**). The results of KEGG enrichment analysis revealed that DEGs were significantly enriched in the NOD-like receptor signaling pathway and the NF-kappa B signaling pathway (**Figure 4B**; **Supplementary Table 4**).

### 3.6 Identification of two distinct gene patterns

**Figure 5:**
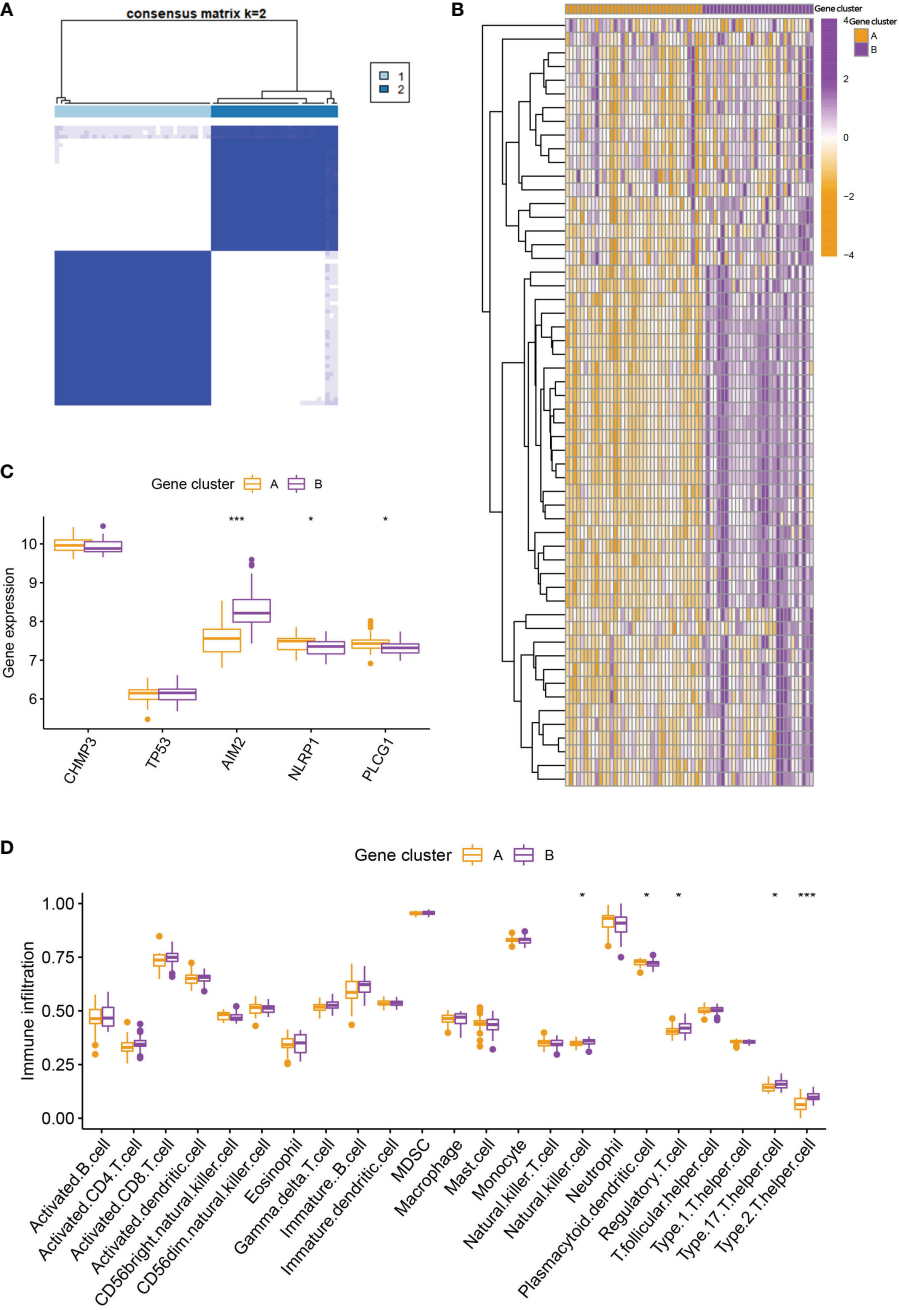
Consensus clustering of the DEGs. **(A)** Consensus matrices of the 56 DEGs for k = 2. **(B)** Expression heatmap of the 56 DEGs in gene cluster A and B. **(C)** Differential expression histogram of the 5 PRGs in gene cluster A and B. **(D)** Differential immune cell infiltration between gene cluster A and B. * means P < 0.05, *** means P < 0.001.

**Figure 6:**
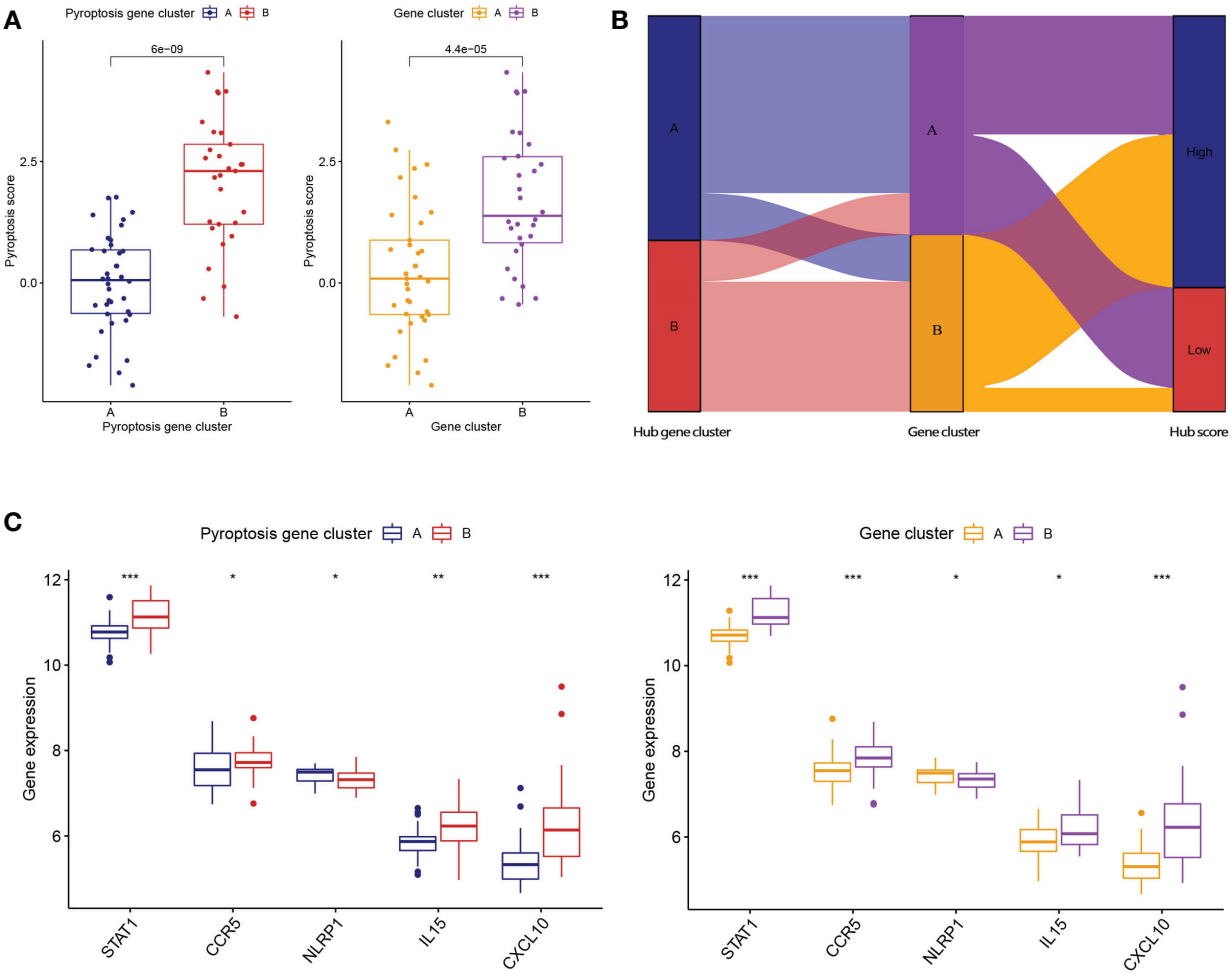
Role of pyroptosis patterns in distinguishing RA. **(A)** Differences in pyroptosis score between pyroptosis gene cluster A and B and differences in pyroptosis score between gene cluster A and B. **(B)** Sankey diagram showing the relationship between pyroptosis patterns, pyroptosis gene patterns, and pyroptosis scores. **(C)** Differential expression levels of STAT1, CCR5, NLRP1, IL-15, and CXCL10 between pyroptosis gene cluster A and B. **(C)** Differential expression levels of STAT1, CCR5, NLRP1, IL-15, and CXCL10 between gene cluster A and B. * means P < 0.05, ** means P < 0.01, *** means P < 0.001.

To further verify the pyroptosis patterns, we classified the RA patients into different genetic subtypes and termed as gene cluster A and B based on the 56 DEGs by using the consensus clustering method (**Figure 5A**; **Supplementary Figure 2**). There were 37 cases in gene cluster A and 30 in gene cluster B. As shown in **Figure 5B**, the heatmap displayed the expression levels of the 56 DEGs in gene clusters A and B. In addition, we found that the differential expression levels of the 5 significant PRGs and immune cell infiltration between gene cluster A and B were consistent with those in the pyroptosis patterns (**Figures 5C, D**). The result again demonstrated the accuracy of dividing into distinct subtypes. Furthermore, we also compared the pyroptosis score between the two distinct pyroptosis patterns or DEGs patterns. The result revealed that the pyroptosis score in cluster B or gene cluster B was significantly higher than that in cluster A, or gene cluster A (**Figure 6A**). The relationship between pyroptosis patterns, pyroptosis gene patterns, and pyroptosis scores was visualized in a Sankey diagram (**Figure 6B**).

### 3.7 Identification of two distinct gene patterns

To further reveal the relationship between pyroptosis patterns and RA, we investigated the correlation between pyroptosis patterns and STAT1, CCR5, NLRP1, IL-15, and CXCL10. The results showed that the expression levels of STAT1, CCR5, NLRP1, IL-15, and CXCL10 were higher in pyroptosis gene cluster B or gene cluster B than in pyroptosis gene cluster A or gene cluster A, which suggested that pyroptosis gene cluster B or gene cluster B is highly linked to RA characterized by the immune response **Figure 6C**.

## 4 Discussion

RA is a chronic inflammatory disease characterized by persistent inflammatory synovitis and systemic inflammation. RA has attracted wide world attention in recent years due to its high disability rate (32). Currently, treatment strategies with biologics and disease-modifying anti-rheumatic drugs have led to significant improvement in the prognosis of RA patients, while a large proportion of RA patients still do not experience effective clinical relief. Studies showed that early diagnosis and positive treatment significantly improve the clinical prognosis of RA (33). Thus, there is an urgent need to identify RA-related diagnostic genes, further explore the molecular mechanisms of RA, and provide novel therapeutic strategies for the prevention and treatment of RA. Pyroptosis is a novel form of inflammatory programmed cell death that plays a vital role in the development of RA (34). Pyroptosis further exacerbates RA inflammation by releasing inflammatory cytokines like interleukin (IL)-1β and IL-18 (35). Besides, studies demonstrated that the serum concentrations of IL-1β (36) and IL-18 (37) were significantly higher in RA patients compared to healthy controls. In order to gain new knowledge for the diagnosis and management of RA, we further studied the connection between RA and pyroptosis by locating and screening PRGs in the serum of RA patients.

In this work, we used 51 PRGs to detect differential expression PRGs using differential expression analysis. We chose 5 candidate PRGs (CHMP3, TP53, AIM2, NLRP1, and PLCG1) from differential expression PRGs by applying RF, SVM-RFE, and LASSO methods in order to filter the 51 PRGs that were the most pertinent for RA. Then, we constructed a nomogram model based on the 5 PRGs to predict the occurrence of RA. In addition, we distinguished two different pyroptosis regulation patterns based on the 5 PRGs and explored the correlation between infiltrating immune cells and the 5 PRGs. A total of 56 DEGs were screened between the two pyroptosis patterns. We further investigated the GO and KEGG functional enrichment of 56 DEGs. Furthermore, we used the consensus clustering method to validate the pyroptosis patterns based on 56 DEGs. We found that two distinct pyroptosis gene patterns were consistent with the grouping of pyroptosis patterns. During the progression of RA, cytokines have been involved in immune regulation, immune response, and inflammatory response (38). We also explore the relationship between inflammatory cytokines and the patterns of pyroptosis.

NOD-like receptor thermal protein domain associated protein 1 (NLRP1) is a member of the NLR family. NLRP1 has been found to be closely associated with the pathogenesis of RA (39). Activated NLRP1 promoted the release of inflammatory cytokines, such as IL-1β and IL-18 (40). Besides, a study showed that inhibition of NLRP1 activation effectively ameliorated joint inflammation and destruction in collagen-induced arthritis mice (41). Furthermore, the polymorphism of the NLRP1 gene was associated with the incidence of RA in the Han Chinese population (42). A member of the interferon-inducible HIN-200 protein family is absent in melanoma 2 (AIM2). AIM2 has emerged as a hub for research into the pyroptosis-specific pathophysiology of RA. AIM2 has been linked to the emergence of inflammatory illnesses and autoimmune arthritis, according to a research (43). AIM2 could format a caspase-1-activating inflammasome, thereby controlling the proteolytic maturation of pro-inflammatory cytokines IL-1β and IL-18 (44). In addition, a meta-analysis revealed that AIM2 levels were highly expressed in peripheral blood mononuclear cells from RA patients (45). Recent study showed that the expression of AIM2 was higher in the RA synovium than in the OA. AIM2 siRNA could inhibit the proliferation of RA fibroblast-like synoviocytes (46).

PLCG1, also called phospholipase C, gamma 1, is involved in the receptor tyrosine kinase-(RTK-)-mediated signal transduction pathway (47). A study found that PRGPI might serve as a prognostic biomarker for pancreatic cancer patients (48). Besides, numerous studies have proven the involvement of PLCG1-mediated inflammatory response in the pathogenesis of osteoarthritis and lung cancer (49, 50). Charged multivesicular body protein 3 (CHMP3) is a subunit of ESCRT III involved in membrane remodeling (51). High CHMP3 expression in breast cancer patients predicts better survival outcomes (52). Moreover, immunohistochemistry revealed significant high expression of CHMP3 in tumor liver tissue (53). The P53 tumor suppressor gene (TP53), also known as the p53 gene, is a protein encoding a molecular weight of 53 kDa. TP53 was found to regulate important cellular functions, such as apoptosis, cell cycle regulation, DNA repair, and apoptosis (54). Besides, TP53 is an inflammatory suppressor associated with autoimmune diseases. Many studies have indicated that the TP53 mutation is closely related to the pathological changes of RA (55, 56). TP53 mutation was identified in synovium of RA patients (57). In the collagen-induced arthritis model, p53**^-/-^** mice showed increased severity of arthritis (58).

However, there are some limits to the study. Firstly, the lack of experimental verification of bioinformatics analysis results. We need to collect human serum samples to further validate our analysis results and elucidate their value as potential clinical biomarkers. Besides, due to the small number of available RA datasets in the GEO database and the limited sample size of this study, the analysis results may be biased. We will include more samples to further assess the reliability of the predicted signature genes.

## 5 Conclusion

In conclusion, our study first found PLCG1 and CHMP3 may be involved in the pathogenesis of RA. And pyroptosis pattern is involved in the progress of RA by bioinformatics analysis, which provides a novel prospective for the prevention and diagnosis of RA.

## Data availability statement

The datasets presented in this study can be found in online repositories. The names of the repository/repositories and accession number(s) can be found in the article/**Supplementary Material**.

## Author contributions

JZ, JGuo, and HZ contributed to the study design and critical revision of the manuscript. JL carried out the study and drafted the manuscript. JL, YC, XJ, DH, XC, HR, and JGuo analyzed the data. All authors read and approved the final manuscript.

## Supplementary material

Supplementary Figure 1 Consensus clustering of the 5 PRGs in RA. (A-G) Consensus matrices of the 5 PRGs for k = 3–9.

Supplementary Figure 2 Consensus clustering of the 56 DEGs in RA. (A-G) Consensus matrices of the 56 DEGs for k = 3–9.

Supplementary Table 1 The list of 52 PRGs.

Supplementary Table 2 The details of PRG screened by the LASSO, RF, and SVM-RFE algorithms.

Supplementary Table 3 The details of GO analysis.

Supplementary Table 4 The details of KEGG analysis.

## References

[1] Ferreira-Silva M Faria-Silva C Viana BP Fernandes E Ramos FA Corvo ML. Liposomal nanosystems in rheumatoid arthritis. Pharmaceutics (2021) 13(4):454. doi: 10.3390/pharmaceutics13040454 PMC8065723 33801603

[2] Firestein GS McInnes IB. Immunopathogenesis of rheumatoid arthritis. Immunity (2017) 46:183–96. doi: 10.1016/j.immuni.2017.02.006 PMC5385708 28228278

[3] Ishikawa Y Terao C. The impact of cigarette smoking on risk of rheumatoid arthritis: A narrative review. Cells-basel (2020) 9(2):475. doi: 10.3390/cells9020475 PMC7072747 32092988

[4] Aletaha D Smolen JS. Diagnosis and management of rheumatoid arthritis: A review. JAMA (2018) 320:1360–72. doi: 10.1001/jama.2018.13103 30285183

[5] Shi J Gao W Shao F. Pyroptosis: Gasdermin-mediated programmed necrotic cell death. Trends Biochem Sci (2017) 42(4):245–54. doi: 10.1016/j.tibs.2016.10.004 27932073

[6] Tsuchiya K. Inflammasome-associated cell death: Pyroptosis, apoptosis, and physiological implications. Microbiol Immunol (2020) 64:252–69. doi: 10.1111/1348-0421.12771 31912554

[7] Liang F Zhang F Zhang L Wei W. The advances in pyroptosis initiated by inflammasome in inflammatory and immune diseases. Inflammation Res (2020) 69:159–66. doi: 10.1007/s00011-020-01315-3 31932850

[8] Vande Walle L Van Opdenbosch N Jacques P Fossoul A Verheugen E Vogel P. Negative regulation of the NLRP3 inflammasome by A20 protects against arthritis. Nature (2014) 512:69–73. doi: 10.1038/nature13322 25043000 PMC4126806

[9] Tan G Huang C Chen J Chen B Zhi F. Gasdermin-e-mediated pyroptosis participates in the pathogenesis of crohn’s disease by promoting intestinal inflammation. Cell Rep (2021) 35:109265. doi: 10.1016/j.celrep.2021.109265 34133932

[10] Zhuang J Cui H Zhuang L Zhai Z Yang F Luo G. Bronchial epithelial pyroptosis promotes airway inflammation in a murine model of toluene diisocyanate-induced asthma. Biomed pharmacother = Biomed pharmacother (2020) 125:109925. doi: 10.1016/j.biopha.2020.109925 32014690

[11] Tasaki S Suzuki K Kassai Y Takeshita M Murota A Kondo Y. Multi-omics monitoring of drug response in rheumatoid arthritis in pursuit of molecular remission. Nat Commun (2018) 9:2755. doi: 10.1038/s41467-018-05044-4 30013029 PMC6048065

[12] Gautier L Cope L Bolstad BM Irizarry RA. Affy–analysis of affymetrix GeneChip data at the probe level. Bioinformatics (2004) 20:307–15. doi: 10.1093/bioinformatics/btg405 14960456

[13] Xing Z Liu Z Fu X Zhou S Liu L Dang Q. Clinical significance and immune landscape of a pyroptosis-derived LncRNA signature for glioblastoma. Front Cell Dev Biol (2022) 10:805291. doi: 10.3389/fcell.2022.805291 35223836 PMC8866949

[14] Zeng R Huang S Qiu X Zhuo Z Wu H Jiang L. Predicting the prognosis of esophageal adenocarcinoma by a pyroptosis-related gene signature. Front Pharmacol (2021) 12:767187. doi: 10.3389/fphar.2021.767187 34867395 PMC8637127

[15] Ding J He X Luo W Zhou W Chen R Cao G. Development and validation of a pyroptosis-related signature for predicting prognosis in hepatocellular carcinoma. Front Genet (2022) 13:801419. doi: 10.3389/fgene.2022.801419 35140750 PMC8818951

[16] Tibshirani R. Least squares support vector machine classifiers. J R Stat Society Ser B (Methodological) (1996) 58:267–88. doi: 10.1111/j.2517-6161.1996.tb02080.x

[17] Suykens JAK VJ. Regression shrinkage and selection via the lasso. Neural Process Lett (1999) 9:293–300. doi: 10.1023/A:1018628609742

[18] Strobl C Boulesteix AL Zeileis A Hothorn T. Bias in random forest variable importance measures: illustrations, sources and a solution. BMC Bioinf (2007) 8:25. doi: 10.1186/1471-2105-8-25 PMC1796903 17254353

[19] Huang ML Hung YH Lee WM Li RK Jiang BR. SVM-RFE based feature selection and taguchi parameters optimization for multiclass SVM classifier. ScientificWorldJournal (2014) 2014:795624. doi: 10.1155/2014/795624 25295306 PMC4175386

[20] Friedman J Hastie T Tibshirani R. Regularization paths for generalized linear models *via* coordinate descent. J Stat Softw (2010) 33:1–22. 20808728 PMC2929880

[21] Cutler A Stevens JR. Random forests for microarrays. Methods Enzymol (2006) 411:422–32. doi: 10.1016/S0076-6879(06)11023-X 16939804

[22] Iasonos A Schrag D Raj GV Panageas KS. How to build and interpret a nomogram for cancer prognosis. J Clin Oncol (2008) 26:1364–70. doi: 10.1200/JCO.2007.12.9791 18323559

[23] Vickers AJ Elkin EB. Decision curve analysis: a novel method for evaluating prediction models. Med Decis Making (2006) 26:565–74. doi: 10.1177/0272989X06295361 PMC2577036 17099194

[24] Tanzhu G Li N Li Z Zhou R Shen L. Molecular subtypes and prognostic signature of pyroptosis-related lncRNAs in glioma patients. Front Oncol (2022) 12:779168. doi: 10.3389/fonc.2022.779168 35237509 PMC8884250

[25] Wilkerson MD Hayes DN. ConsensusClusterPlus: a class discovery tool with confidence assessments and item tracking. Bioinformatics (2010) 26:1572–3. doi: 10.1093/bioinformatics/btq170 PMC2881355 20427518

[26] Meng J Huang X Qiu Y Zheng X Huang J Wen Z. Pyroptosis-related gene mediated modification patterns and immune cell infiltration landscapes in cutaneous melanoma to aid immunotherapy. Aging (Albany NY) (2021) 13:24379–401. doi: 10.18632/aging.203687 PMC8610130 34753832

[27] Wu J Zhu Y Luo M Li L. Comprehensive analysis of pyroptosis-related genes and tumor microenvironment infiltration characterization in breast cancer. Front Immunol (2021) 12:748221. doi: 10.3389/fimmu.2021.748221 34659246 PMC8515898

[28] Hänzelmann S Castelo R Guinney J. GSVA: gene set variation analysis for microarray and RNA-seq data. BMC Bioinf (2013) 14:7. doi: 10.1186/1471-2105-14-7 PMC3618321 23323831

[29] Charoentong P Finotello F Angelova M Mayer C Efremova M Rieder D. Pan-cancer immunogenomic analyses reveal genotype-immunophenotype relationships and predictors of response to checkpoint blockade. Cell Rep (2017) 18:248–62. doi: 10.1016/j.celrep.2016.12.019 28052254

[30] Yu G Wang L Han Y He Q. clusterProfiler: an r package for comparing biological themes among gene clusters. OMICS: A J Integr Biol (2012) 16:284–7. doi: 10.1089/omi.2011.0118 PMC3339379 22455463

[31] Huang DW Sherman BT Lempicki RA. Bioinformatics enrichment tools: paths toward the comprehensive functional analysis of large gene lists. Nucleic Acids Res (2009) 37:1–13. doi: 10.1093/nar/gkn923 19033363 PMC2615629

[32] Bai L Zhang Y Wang P Zhu X Xiong JW Cui L. Improved diagnosis of rheumatoid arthritis using an artificial neural network. Sci Rep (2022) 12:9810. doi: 10.1038/s41598-022-13750-9 35697754 PMC9192742

[33] Wu H Wu H He Y Gan Z Xu Z Zhou M. Synovitis in mice with inflammatory arthritis monitored with quantitative analysis of dynamic contrast-enhanced NIR fluorescence imaging using iRGD-targeted liposomes as fluorescence probes. Int J Nanomed (2018) 13:1841–50. doi: 10.2147/IJN.S155475 PMC5870656 29615837

[34] Kong R Sun L Li H Wang D. The role of NLRP3 inflammasome in the pathogenesis of rheumatic disease. Autoimmunity (2022) 55:1–7. doi: 10.1080/08916934.2021.1995860 34713773

[35] Zhao J Jiang P Guo S Schrodi SJ He D. Apoptosis, autophagy, NETosis, necroptosis, and pyroptosis mediated programmed cell death as targets for innovative therapy in rheumatoid arthritis. Front Immunol (2021) 12:809806. doi: 10.3389/fimmu.2021.809806 35003139 PMC8739882

[36] Verma I Syngle A Krishan P. Predictors of endothelial dysfunction and atherosclerosis in rheumatoid arthritis in Indian population. Indian Heart J (2017) 69:200–6. doi: 10.1016/j.ihj.2016.10.013 PMC5414984 28460767

[37] Min HK Kim S Lee JY Kim KW Lee SH Kim HR. IL-18 binding protein suppresses IL-17-induced osteoclastogenesis and rectifies type 17 helper T cell/regulatory T cell imbalance in rheumatoid arthritis. J Transl Med (2021) 19:392. doi: 10.1186/s12967-021-03071-2 34530864 PMC8444577

[38] Guo Q Mao X Zhang Y Meng S Xi Y Ding Y. Guizhi-Shaoyao-Zhimu decoction attenuates rheumatoid arthritis partially by reversing inflammation-immune system imbalance. J Transl Med (2016) 14:165. doi: 10.1186/s12967-016-0921-x 27277474 PMC4898408

[39] Evavold CL Kagan JC. How inflammasomes inform adaptive immunity. J Mol Biol (2018) 430:217–37. doi: 10.1016/j.jmb.2017.09.019 PMC5766381 28987733

[40] Addobbati C Da Cruz H Adelino JE Melo Tavares Ramos AL Fragoso TS Domingues A. Polymorphisms and expression of inflammasome genes are associated with the development and severity of rheumatoid arthritis in Brazilian patients. Inflammation res: Off J Eur Histamine Res Soc (2018) 67:255–64. doi: 10.1007/s00011-017-1119-2 29230505

[41] Li F Guo N Ma Y Ning B Wang Y Kou L. Inhibition of P2X4 suppresses joint inflammation and damage in collagen-induced arthritis. Inflammation (2014) 37:146–53. doi: 10.1007/s10753-013-9723-y 24062058

[42] Sui J Li H Fang Y Liu Y Li M Zhong B. NLRP1 gene polymorphism influences gene transcription and is a risk factor for rheumatoid arthritis in han chinese. Arthritis Rheum (2012) 64:647–54. doi: 10.1002/art.33370 21976003

[43] Man SM Karki R Kanneganti TD. AIM2 inflammasome in infection, cancer, and autoimmunity: Role in DNA sensing, inflammation, and innate immunity. Eur J Immunol (2016) 46:269–80. doi: 10.1002/eji.201545839 PMC4758349 26626159

[44] Baum R Sharma S Carpenter S Li QZ Busto P Fitzgerald KA. Cutting edge: AIM2 and endosomal TLRs differentially regulate arthritis and autoantibody production in DNase II-deficient mice. J Immunol (2015) 194:873–7. doi: 10.4049/jimmunol.1402573 PMC4299698 25548216

[45] Afroz S Giddaluru J Vishwakarma S Naz S Khan AA Khan N. A comprehensive gene expression meta-analysis identifies novel immune signatures in rheumatoid arthritis patients. Front Immunol (2017) 8:74. doi: 10.3389/fimmu.2017.00074 28210261 PMC5288395

[46] Chen Y Fujuan Q Chen E Yu B Zuo F Yuan Y. Expression of AIM2 in rheumatoid arthritis and its role on fibroblast-like synoviocytes. Mediat Inflamm (2020) 2020:1693730. doi: 10.1155/2020/1693730 PMC7605934 33162829

[47] Zhang G Yan Z. A new definition of pyroptosis-related gene markers to predict the prognosis of lung adenocarcinoma. BioMed Res Int (2021) 2021:8175003. doi: 10.1155/2021/8175003 34869771 PMC8642010

[48] Xie W Li X Yang C Li J Shen G Chen H. The pyroptosis-related gene prognostic index associated with tumor immune infiltration for pancreatic cancer. Int J Mol Sci (2022) 23(11):6178. doi: 10.3390/ijms23116178 35682857 PMC9180955

[49] Kim MS Yang YM Son A Tian YS Lee SI Kang SW. RANKL-mediated reactive oxygen species pathway that induces long lasting Ca2+ oscillations essential for osteoclastogenesis. J Biol Chem (2010) 285:6913–21. doi: 10.1074/jbc.M109.051557 PMC2844141 20048168

[50] Song W Kim LC Han W Hou Y Edwards DN Wang S. Phosphorylation of PLCgamma1 by EphA2 receptor tyrosine kinase promotes tumor growth in lung cancer. Mol Cancer Res (2020) 18:1735–43. doi: 10.1158/1541-7786.MCR-20-0075 PMC7641970 32753469

[51] Di Giaimo R Penna E Pizzella A Cirillo R Perrone-Capano C Crispino M. Cross talk at the cytoskeleton-plasma membrane interface: Impact on neuronal morphology and functions. Int J Mol Sci (2020) 21(23):9133. doi: 10.3390/ijms21239133 33266269 PMC7730950

[52] Wang Z Wang X. miR-122-5p promotes aggression and epithelial-mesenchymal transition in triple-negative breast cancer by suppressing charged multivesicular body protein 3 through mitogen-activated protein kinase signaling. J Cell Physiol (2020) 235:2825–35. doi: 10.1002/jcp.29188 31541468

[53] Guo Y Shang A Wang S Wang M. Multidimensional analysis of CHMP family members in hepatocellular carcinoma. Int J Gen Med (2022) 15:2877–94. doi: 10.2147/IJGM.S350228 PMC8923641 35300135

[54] Wang H Wang X Xu L Zhang J. TP53 and TP53-associated genes are correlated with the prognosis of paediatric neuroblastoma. BMC Genom Data (2022) 23:41. doi: 10.1186/s12863-022-01059-5 35655142 PMC9164562

[55] Gansmo LB Lie BA Maehlen MT Vatten L Romundstad P Hveem K. Polymorphisms in the TP53-MDM2-MDM4-axis in patients with rheumatoid arthritis. Gene (2021) 793:145747. doi: 10.1016/j.gene.2021.145747 34077778

[56] Kato S Lippman SM Flaherty KT Kurzrock R. The conundrum of genetic “Drivers” in benign conditions. J Natl Cancer Institute (2016) 108(8):djw036. doi: 10.1093/jnci/djw036 PMC5017937 27059373

[57] Bolivar AM Luthra R Mehrotra M Chen W Barkoh BA Hu P. Targeted next-generation sequencing of endometrial cancer and matched circulating tumor DNA: identification of plasma-based, tumor-associated mutations in early stage patients. Mod Pathol (2019) 32:405–14. doi: 10.1038/s41379-018-0158-8 PMC6395490 30315273

[58] Yamanishi Y Boyle DL Pinkoski MJ Mahboubi A Lin T Han Z. Regulation of joint destruction and inflammation by p53 in collagen-induced arthritis. Am J Pathol (2002) 160:123–30. doi: 10.1016/S0002-9440(10)64356-8 PMC1867134 11786406

